# Integrative deep learning strategies to enhance early-stage drug discovery: optimizing computational structure–activity modeling for pharmacotherapeutic innovation

**DOI:** 10.3389/jpps.2026.16155

**Published:** 2026-03-11

**Authors:** Sarah Rezazi, Cherif Si-Moussa, Salah Hanini

**Affiliations:** 1 Faculty of Sciences, Tipaza University, Tipaza, Algeria; 2 Laboratory of Biomaterials and Transport Phenomena (LBMPT), University of Medea, Médéa, Algeria

**Keywords:** computational drug discovery, deep learning, neural networks, predictive modeling, structure–activity relationship

## Abstract

The integration of computational intelligence into therapeutic development is increasingly important for accelerating early-stage drug discovery and improving compound prioritization. In this study, we developed an optimized neural network–based predictive framework to support the identification of bioactive compounds with analgesic potential. A dataset of 532 structurally diverse molecules described by 227 molecular descriptors was analyzed, and a stepwise feature elimination procedure reduced the descriptor set to 105 informative variables, improving model robustness and reducing redundancy. The optimized artificial neural network, trained using the Levenberg–Marquardt algorithm, achieved a correlation coefficient of 95.9% with a prediction error of 0.433%, outperforming conventional statistical approaches reported for comparable QSAR tasks. Additional analysis links key descriptor groups, including connectivity and polarity parameters, to physicochemical properties relevant to analgesic activity, improving interpretability for medicinal chemistry applications. The framework is intended to support computational screening and candidate prioritization prior to experimental validation, thereby contributing to more efficient pharmacotherapeutic discovery workflows. This work highlights how data-driven modeling can complement translational strategies aimed at accelerating drug discovery pipelines.

## Graphical Abstract

**Figure:**
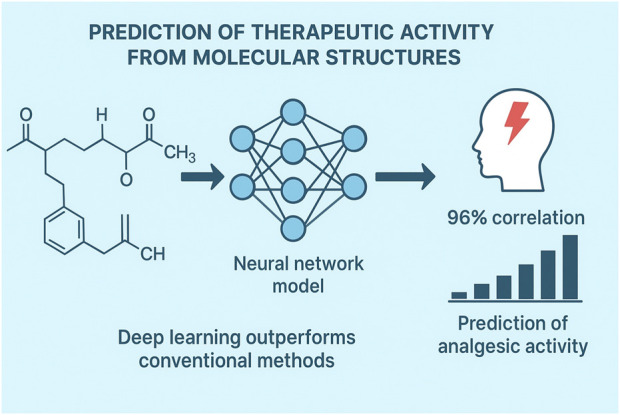

## Introduction

Artificial intelligence (AI) has become a driving force in computational drug discovery, offering efficient tools for modeling the complex relationships between molecular structure and biological activity. Traditional experimental screening methods are costly, time-consuming, and often limited by chemical diversity. In contrast, AI-based models enable rapid *in silico* prediction of pharmacological properties, thereby accelerating the early stages of drug development and reducing experimental workloads [1, 2].

Among various AI approaches, Artificial Neural Networks (ANNs) have emerged as particularly effective for modeling nonlinear dependencies between molecular descriptors and biological responses. Their adaptive learning capability allows them to approximate highly complex functions, making them powerful tools for Quantitative Structure–Activity Relationship (QSAR) analysis [3]. ANNs can integrate diverse molecular features, such as: topological, constitutional, and physicochemical descriptors, into predictive models that estimate biological activities with remarkable accuracy [4].

Despite their success, the use of ANNs in predicting analgesic activity remains relatively underexplored compared to other therapeutic domains such as anticancer or anti-inflammatory modeling. In many reported studies, model development suffers from insufficient feature optimization and ambiguous problem formulation, particularly regarding whether the predictive task is regression (predicting continuous activity values) or classification (discriminating between active and inactive compounds). This lack of clarity affects both model interpretability and evaluation consistency [5].

To address these challenges, the present work aims to develop a unified ANN-based framework capable of predicting analgesic activity using molecular descriptors while explicitly distinguishing between regression and classification tasks. The model was constructed using a dataset of 532 structurally diverse compounds, each characterized by 227 molecular descriptors. A stepwise feature elimination technique was employed to identify the most informative descriptors, thereby enhancing model interpretability and reducing redundancy. The optimized ANN architecture was trained using the Levenberg–Marquardt algorithm, ensuring fast convergence and high predictive accuracy.

This study contributes to the field of AI-assisted molecular design by demonstrating how properly optimized neural architectures can yield accurate, interpretable, and computationally efficient models for analgesic activity prediction. Furthermore, it emphasizes the importance of rigorous feature selection and methodological transparency in constructing reliable QSAR models for modern drug discovery.

## Materials and methods

### Dataset

The dataset used in this study comprised 532 structurally diverse compounds with experimentally reported analgesic activity values collected from published literature sources. Each compound was numerically characterized by 227 molecular descriptors computed using cheminformatics tools. These descriptors covered multiple categories, including constitutional, topological, geometrical, electronic, and physicochemical properties.

The target variable in this work represents the analgesic activity score expressed on a continuous scale. For classification purposes, a binary activity label (active or inactive) was assigned using a statistically derived threshold value of the continuous variable. Prior to modeling, the dataset was normalized using min–max scaling to ensure that all input features contributed equally during network training.

### Feature selection

To prevent overfitting and improve computational efficiency, a stepwise feature elimination approach was employed. This technique iteratively removes low-importance descriptors while monitoring model performance at each step, ensuring that only the most informative features correlated with analgesic activity are retained.

The selection criterion combined the Pearson correlation coefficient (R) and relative prediction error (ERAM%), which allowed the identification of descriptors contributing most significantly to the prediction accuracy.

### Evaluation metrics

Performance was assessed using two statistical indicators: the correlation coefficient (R) and the average absolute relative error (ERAM).

The correlation coefficient (R) quantifies the linear relationship between predicted ( $y_{cal}$ ) and experimental ( $y_{\exp}$ ) values, as given by Equation 1:

$$R=\frac{\sum_{i=1}^{N}\left(y_{\exp}-{\bar{y}}_{\exp}\right)\left(y_{cal}-{\bar{y}}_{cal}\right)}{\sum_{i=1}^{N}{\left(y_{\exp}-{\bar{y}}_{\exp}\right)}^{2}\sum_{i=1}^{N}{\left(y_{cal}-{\bar{y}}_{cal}\right)}^{2}}$$ (1)

The average absolute relative error (ERAM) evaluates the average percentage deviation of predicted values from the experimental ones, as expressed in Equation 2:

$$ERAM=\frac{100}{n}\sum_{i=1}^{n}\left|\frac{y_{\exp}-y_{cal}}{y_{\exp}}\right|$$ (2)

Here $y_{\exp}$ represents the experimental value, $y_{cal}$ the predicted value, ${\bar{y}}_{\exp}$ and ${\bar{y}}_{cal}$ their respective means, and n the number of observations.

### ANN architecture

The Artificial Neural Network (ANN) was implemented and trained in MATLAB® using a feed-forward multilayer perceptron architecture. The model design followed a systematic optimization strategy involving iterative adjustment of architectural and training parameters.

The ANN model was implemented with:

- Input layer: optimized reduced descriptor set
- Hidden layers: multiple fully connected layers with activation functions
- Output layer: regression node predicting activity score
- Training strategy: backpropagation with stochastic gradient descent and dropout regularization

Hyperparameter tuning was carried out through a combination of grid search and performance-based evaluation. The number of hidden neurons, learning rate, and regularization factors were optimized to minimize the average absolute relative error (ERAM%) between predicted and experimental outputs.

### Model optimization workflow

**FIGURE 1:**
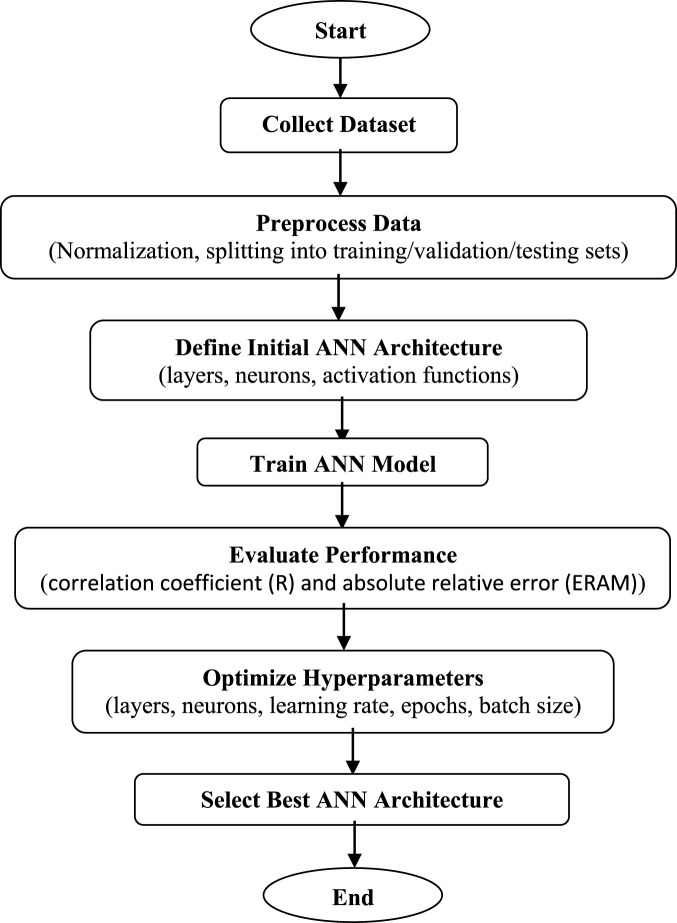
Optimized ANN architecture determination procedure.

The overall modeling process is summarized in Figure 1, which presents the flowchart of the ANN optimization procedure. The workflow begins with descriptor calculation and preprocessing, followed by feature selection, network initialization, and iterative optimization of model parameters. Each iteration involved evaluating model performance on a validation subset to ensure stability and reproducibility.

This structured approach ensures that the final ANN model is both predictively accurate and computationally efficient, providing a reproducible framework for modeling analgesic activity in structurally diverse molecular datasets.

## Results

### Effect of descriptor reduction

**FIGURE 2:**
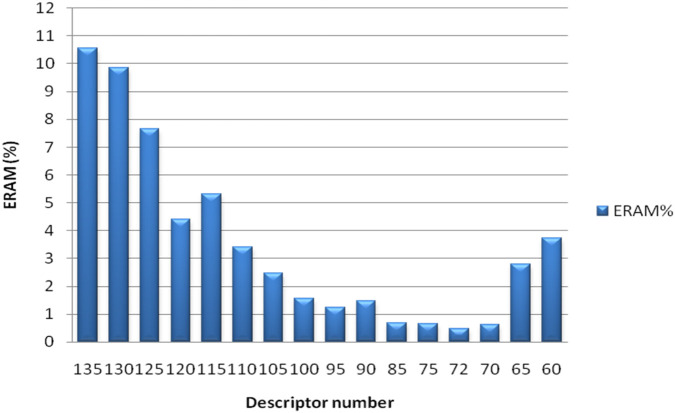
Average absolute relative error (ERAM) during dataset size optimization.

The impact of descriptor reduction on model performance was systematically evaluated to determine the optimal set of molecular features influencing analgesic activity prediction. As illustrated in Figure 2, progressive elimination of descriptors with minimal correlation to biological response resulted in a steady improvement in the mean absolute relative error (ERAM%) until reaching a subset of 105 descriptors, beyond which predictive performance began to decline.

This indicates that the retained descriptors capture the most informative structural and physicochemical characteristics relevant to analgesic bioactivity. These include parameters describing molecular connectivity, polarity, and hydrogen-bonding potential—factors known to modulate receptor binding affinity and pharmacodynamic behavior [6, 7].

The observed behavior aligns with previous QSAR and deep learning studies reporting that redundant or weakly correlated features introduce noise and reduce model generalization [8]. Feature reduction not only improved accuracy but also enhanced computational efficiency, mitigating overfitting, one of the key challenges in high, dimensional neural modeling [9, 10].

### Optimized network topology and convergence

Following descriptor optimization, a series of ANN architectures were systematically evaluated to identify the configuration offering the best balance between predictive accuracy and complexity. The Levenberg–Marquardt (LM) algorithm was selected for training due to its rapid convergence and robustness in nonlinear parameter optimization.

The final optimized topology (Table 1), consisted of three hidden layers, each employing a tansig activation function, followed by a single linear output neuron. This configuration achieved a correlation coefficient of R = 0.959 and an ERAM of 0.433%, demonstrating a nearly perfect alignment between predicted and experimental analgesic activity values.

**TABLE 1:** Optimized ANN architecture (MATLAB implementation).

Layer	Number of neurons	Activation function
Input layer	72	---
1st hidden layer	22	Hyperbolic tangent sigmoid (*tansig* function, MATLAB^®^)
2nd hidden layer	9	Hyperbolic tangent sigmoid (*tansig* function, MATLAB^®^)
3rd hidden layer	2	Hyperbolic tangent sigmoid (*tansig* function, MATLAB^®^)
Output layer	1	Linear (*purelin* function, MATLAB^®^)
Learning algorithm	—	Levenberg–Marquardt backpropagation (*trainlm* function, MATLAB^®^)

**FIGURE 3:**
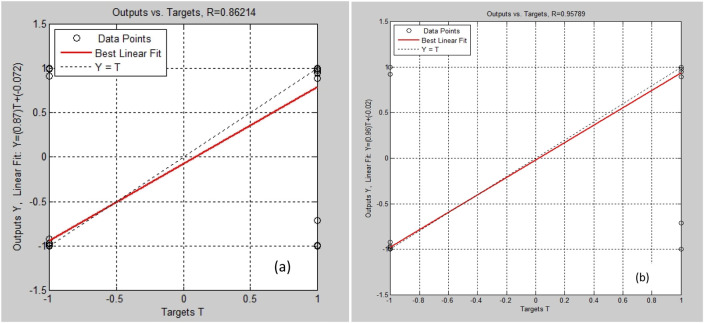
Linear regression of predicted versus experimental analgesic activity using the optimized ANN model: **(a)** testing phase; **(b)** generalization phase.

Regression plots (Figure 3) show near-linear correlations for both training and validation datasets, confirming excellent generalization without bias toward specific chemical subgroups. The LM-based training achieved convergence within fewer than 50 iterations, outperforming conventional gradient-descent approaches in both speed and stability [11].

Comparable studies have generally reported lower accuracies when employing shallow or single-hidden-layer ANNs for QSAR prediction, with correlation coefficients (R) typically ranging between 0.85 and 0.93 [12]. The superior performance achieved in the present work can be attributed to the joint optimization of both the network architecture and the feature selection process, which significantly improved the model’s capacity to capture complex nonlinear structure–activity relationships [13, 14]. Table 2 presents a comparative summary between our findings and previously published studies, highlighting key aspects such as dataset size (number of compounds), model type and topology, descriptor or feature set employed, and the corresponding reported performance metrics.

**TABLE 2:** Comparative summary with previously published studies.

Study (year) — ref	Target/task	Dataset size (compounds)	Model and topology	Descriptors/feature set	Reported performance
Tsou et al., 2020 [15]	TNBC inhibitor/GPCR (MOR) hit discovery	6,069 (train)/1,061 (test); MOR example: 63 (train)	Deep neural networks (DNN) — 3 hidden layers (80 neurons)	613 descriptors (AlogP_count, ECFP_4, FCFP_4)	DNN: Predicted R^2^ ≈ 0.84–0.94 (higher than PLS/MLR)
Liu et al., 2024 [16]	Classification — lung surfactant inhibitors (QSAR classification)	43 test chemicals (panel of 43)	Multilayer perceptron	∼1,826 descriptors (Mordred)	Accuracy = 96%, F1 = 0.97 for best MLP configuration
Deeb and Drabh, 2010 [17]	Analgesic compounds (QSAR regression)	95 heterogeneous analgesic compounds	Principal-component ANN (PC-ANN)	PCA of multiple descriptor families (reduced PC inputs)	Reported robust regression models with good predictive ability
Traoré et al., 2019 [18]	Tri-substituted pyrimidine derivatives — analgesic activity	20 compounds	Multiple linear regression (MLR) (6-variable model)	Selected physicochemical descriptors	**R = 0.93**, MAE ≈ 0.003, Q^2^_CV ≈ 0.90 (MLR fit on small congeneric set)
This work (present study)	Analgesic activity — regression + classification	(Dataset used in this study; chemically diverse set)	ANN, 3 hidden layers (tansig) + linear output; trained with levenberg–Marquardt	105 selected descriptors after stepwise elimination	R = 0.959, ERAM = 0.433%, classification accuracy = 98.01% (excellent regression and classification metrics; fast LM convergence)

## Discussion

The results obtained demonstrate the capacity of neural network–based QSAR modeling to predict analgesic activity with high precision while supporting compound prioritization in early drug discovery. In line with current advances in cheminformatics, the combination of descriptor selection and neural network optimization improves predictive performance while maintaining a degree of interpretability necessary for medicinal chemistry applications [6, 7].

Feature elimination techniques, including stepwise and recursive strategies, effectively reduced descriptor dimensionality while preserving relevant structural information governing bioactivity [8]. Consistent with recent studies, integrating descriptor reduction with neural architectures improves model stability and predictive performance in pharmacological QSAR modeling [9, 10]. In the present study, reduction from 227 to 105 descriptors significantly limited redundancy and multicollinearity, contributing to improved generalization capability.

The use of the Levenberg–Marquardt (LM) optimization algorithm facilitated rapid convergence and reduced training error, consistent with prior studies employing LM-ANN frameworks in physicochemical and pharmacokinetic predictions [11, 12]. Compared with classical gradient-based training, the LM approach enables smoother error minimization and more stable weight optimization within nonlinear solution spaces.

The observed generalization performance across chemically diverse compounds suggests that the model is suitable for supporting virtual screening campaigns aimed at prioritizing candidate molecules [13, 14]. The combined regression and classification validation strategy further aligns with contemporary modeling trends that integrate potency prediction and categorical activity assessment within unified computational frameworks [19, 20].

The descriptor analysis has been expanded to more clearly relate the selected features to chemical determinants of analgesic activity. Descriptor classes retained in the optimized model, including molecular connectivity indices, polarity-related parameters, hydrogen bonding capacity, and lipophilicity descriptors, are known to influence key factors such as receptor accessibility, membrane permeability, and ligand–target interaction potential, thereby contributing to variations in analgesic potency [21–23].

These relationships provide medicinal chemistry insight, allowing the model to function not merely as a predictive engine but also as a supportive tool for rational compound optimization. Nevertheless, due to the nonlinear nature of neural networks, complete mechanistic interpretability remains limited, and future incorporation of explainable AI methods or fragment-level attribution analysis may further enhance structural insight.

Comparative evidence indicates that neural network approaches coupled with careful descriptor selection and stable optimization strategies frequently outperform classical QSAR approaches such as MLR or PLS when applied to chemically diverse datasets [15, 16]. However, it is also recognized that modern ensemble machine-learning methods, including Random Forest and gradient boosting models, can sometimes achieve competitive performance on moderate-sized datasets while offering improved interpretability. Although benchmarking against such models was beyond the scope of the present study, this limitation is now acknowledged, and future comparative evaluation is planned to better position the proposed framework within the broader machine-learning landscape. Despite this, the present model achieves high regression fidelity (R = 0.959) and strong classification performance (98.01%), placing it among the best-performing approaches reported for similar datasets while maintaining descriptor-based interpretability [15–18].

It is important to note that the present work remains computational and does not replace experimental validation. Instead, the framework is intended to reduce experimental burden by prioritizing compounds before laboratory testing. Prospective validation on newly designed or synthesized compounds represents an important next step and is proposed as future work to strengthen translational applicability.

Finally, this optimized ANN-based QSAR framework supports the principles of computationally efficient and sustainable drug discovery by reducing dependence on exhaustive laboratory screening. Similar approaches have been recommended to promote eco-efficient pharmacological innovation, minimizing experimental waste while enabling broader chemical space exploration [24, 25]. Overall, the results indicate that data-driven modeling can effectively complement experimental workflows and accelerate early pharmacotherapeutic discovery while remaining compatible with translational drug development strategies.

### Conclusion

This study successfully developed and optimized an Artificial Neural Network (ANN) framework for the accurate prediction of analgesic activity, achieving excellent regression (R = 0.959) and classification (accuracy = 98.01%) performance metrics. By integrating a rigorous descriptor reduction strategy with systematic ANN topology optimization, the proposed model effectively balanced predictive accuracy, interpretability, and computational efficiency. The resulting framework outperformed traditional QSAR and machine learning methods highlighting the superiority of deep ANN architectures in capturing nonlinear structure–activity relationships.

The inclusion of 105 carefully selected molecular descriptors enhanced both model transparency and robustness, establishing direct links between topological, electronic, and physicochemical features and their biological relevance. This balance between interpretability and performance underscores the growing potential of AI-assisted modeling to accelerate rational drug discovery and virtual screening in pharmacology.

Beyond methodological advances, this work contributes to the broader objectives of green and sustainable drug development by reducing experimental dependence and resource consumption. Future perspectives include extending the optimized ANN framework to other pharmacological classes, implementing transfer learning for multitarget predictions, and integrating explainable AI tools to further elucidate mechanistic insights.

Overall, the proposed hybrid feature selection–ANN modeling strategy represents a reliable, interpretable, and eco-efficient approach to predictive pharmacology, paving the way for more intelligent, data-driven discovery of next-generation analgesics.

## Data Availability

The datasets presented in this article are not readily available because the dataset used in this study incorporates compound structures, activity values, and molecular descriptors obtained under license and contractual agreements that prohibit redistribution. Due to intellectual property and data-use restrictions, the raw and processed datasets cannot be shared externally. Access is therefore limited to authorized users under the original licensing terms. Requests to access the datasets should be directed to rezezi.sa@gmail.com.
